# Role of Intestinal Hydrolase in the Absorption of Prenylated Flavonoids Present in Yinyanghuo

**DOI:** 10.3390/molecules16021336

**Published:** 2011-02-01

**Authors:** Yan Chen, Jinyan Wang, Xiaobin Jia, Xiaobin Tan, Ming Hu

**Affiliations:** 1 Key Laboratory of New Drug Delivery System of Chinese Materia Medica, Jiangsu Provincial Academy of Chinese Medicine, 100 Shizi Road, Nanjing 210028, China; E-Mails: ychen202@hotmail.com (Y.C.); wwind924@yahoo.com.cn (J.W.); jakytan2005@hotmail.com (X.T.); 2 Department of Pharmacological and Pharmaceutical Sciences, College of Pharmacy, University of Houston, 1441 Moursund Street, Houston, TX 77030, USA; E-Mail: mhu@uh.edu (M.H.)

**Keywords:** flavonoid, lactase phlorizin hydrolase, metabolite, rat intestine, Yinyanghuo

## Abstract

*Purpose*: Yinyanghuo (Herba Epimdii) is a traditional Chinese herb containing prenylated flavonoids as its active constituents. The aim of this study was to examine the significance of the intestinal hydrolysis of prenylated flavonoids by lactase phlorizin hydrolase (LPH), an enzyme at the brush border membrane of intestinal cells. *Methods*: A four-site perfused rat intestinal model was used. The concentration of the flavonoids of interest and their metabolites in different intestinal segements were analyzed by HPLC, and the apparent permeabilities were calculated. A lactase phlorizin hydrolase inhibitor (gluconolactone) was employed to investigate the mechanism of the intestinal absorption, and the metabolites of the four flavonoids were identified using LC/MS/MS. *Results*: Diglycosides (icariin) or triglycosides (epimedin A, epimedin B, and epimedin C) were hydrolyzed rapidly in duodenum and jejunum producing one or two metabolites, while a monoglycoside (baohuoside I) was absorbed directly. When co-perfused with glucono-lactone, both the hydrolysis of diglycosides and triglycosides were significantly inhibited, with inhibition rates for icariin (62%, 50%, 40%, 46%), epimedin A, (55%, 26%, 21%, 14%); epimedin B (42%, 40%, 74%, 22%), and epimedin C (42%, 40%, 52%, 35%) in duodenum, jejunum, ileum, and colon, respectively. Also the metabolites of icariin, epimedin A, epimedin B, and epimedin C were identified as baohuoside I (one of two), sagittatoside A, sagittatoside B, and 2″-*O*-rhamnosylicariside II, respectively. *Conclusions*: The results showed that lactase phlorizin hydrolase was a major determinant of the intestinal absorption of prenylated flavonoids present in Yinyanghuo.

## 1. Introduction

**Figure 1:**
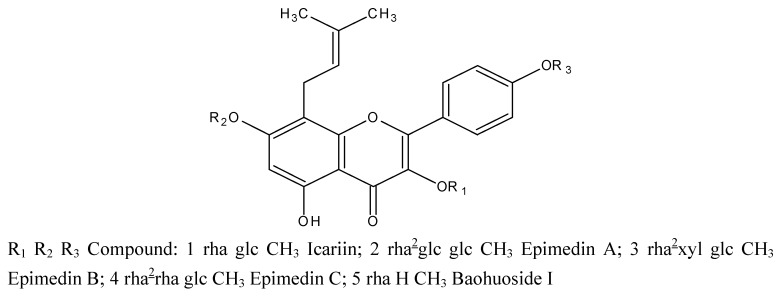
Chemical structures of five prenylated flavonoids isolated from Yinyanghuo or *Epimedium koreamum* Nakai: icariin, epimedin A, epimedin B, epimedin C, and baohuoside I. The symbol “glc” refers to glucose, “rha” to rhamnose, and “xyl” to xylose.

Yinyanghuo (Herba Epimdii,YYH) is widely used as a tonic herbal in traditional Chinese medicine, and is officially listed in the Chinese Pharmacopoeia [1]. Due to its pharmacological actions, it has been used to treat various kinds of disorders such as hypertension, coronary heart disease, osteoporosis, menopause syndrome, breast lump, rheumatism, arthritis and hypogonadism, *etc* [2,3]. The active constituents of the herb are flavonoids, among which prenylated (specifically isopentenyl) flavonoids are the most important. The five main isopentenyl flavonoids present in YYH are diglycosides or triglycosides, namely, icariin, epimedin A, epimedin B, and epimedin C, all of which possess an isopentenyl group at 8-position and a glucose group at the 7-O position and additional sugar moieties at the 3-O position. Another key prenylated flavonoid in YYH is baohuoside I, which only has a rhamnose at the 7-OH position (chemical structures shown in Figure 1) [4].

Recently, it has been reported that the prenylated flavonoids present in the extract of YYH possess anticancer properties [5]. Despite these claims the absorption and metabolism of prenylated flavonoids have not been rigorously examined. Before these flavonoids can exert systemic effects, they must be absorbed from the intestinal lumen, and most of them occur as β--glycosides. It is well known that various glucosidases such as glucocerobrosidase, lactase-phlorizin hydrolase (LPH), cytosolic β-glucosidase (CBG) and pyridoxine glucoside hydrolase are present in the small intestines of human and rats [6]. Amongst them, LPH is the sole β–glucosidase present on the luminal side of the brush border of small intestine and contributes to deglucosylation of the dietary flavonoid glucosides prior to the absorption. Earlier reports have showed that many glucosides, such as apigetrin, genistin, and isoquercetrin can be hydrolyzed by LPH before absorption [7,8,9]. However, limited information is available on prenylated flavonoids present in YYH. Therefore, the objective of the present study was to assess the role of intestinal hydrolase on the absorption of prenylated flavonoid glucosides present in YYH.

In our previous study, the mechanism of *in vitro* absorption of icariin, epimedin A, B, C and baohuoside I have been investigated using the Caco-2 monolayer model [10]. However, the *in vivo* absorption and metabolism of prenylated flavonoids remains unclear as the expression of intestinal enzyme such as LPH is particularly low in Caco-2 cells (EC 3.2.1.23 and 62) [11]. Therefore, here, we used a four-site rat intestinal perfusion model to study the absorptive mechanisms of prenylated flavonoids, because this model is routinely used to investigate drug absorption and metabolism and is recognized by FDA as a viable model of human intestinal absorption. Moreover, this model was also chosen because it has been used previously for the study of flavonoid glycoside, and it was found that the rat intestine expresses large quantities of glucosidases and can rapidly hydrolyze monoglucosides [12,13,14,15].

## 2. Results and Discussion

**Figure 2:**
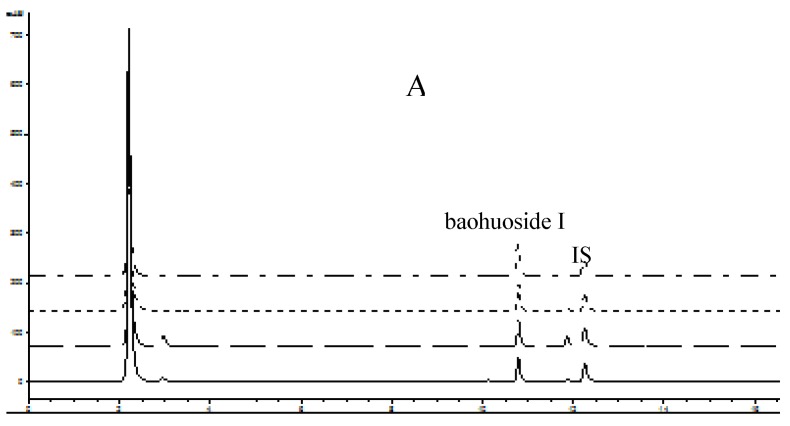

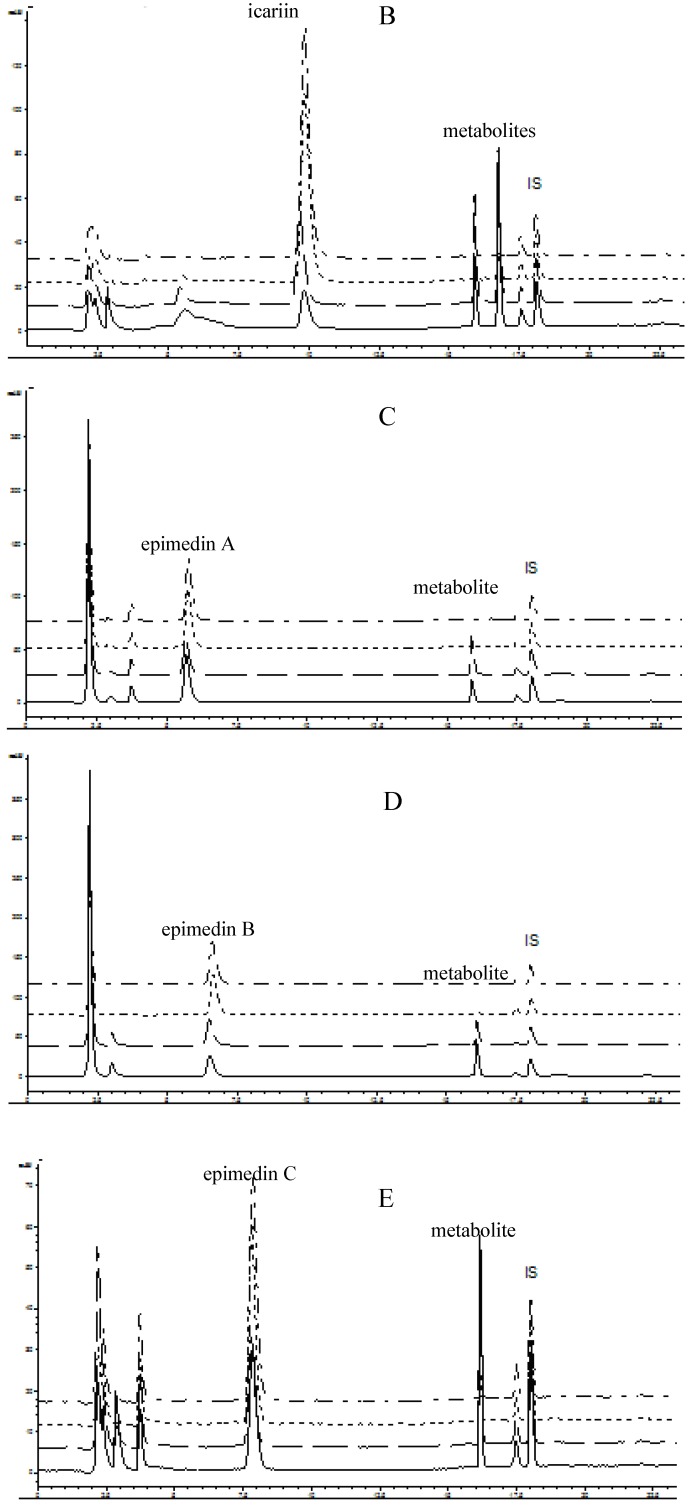
HPLC elution profiles of five prenylflavonoids and their intestinal metabolites as following sequence: duodenum, jejunum, ileum and colon. A(baohuoside I), B(icariin), C(epimedin A), D(epimedin B), E(epimedin C). Testosterone was used as an internal standard (IS).

The HPLC chromatograms of the five prenylated flavonoids are shown in Figure 2. It can be observed from Figure 2A that no metabolites were created from baohuoside I after perfusion, whereas metabolites were generated from icariin, epimedin A, epimedin B, and epimedin C.

For icariin, which is a diglycoside with a glucose group at 7-O position and a rhamnose group at 3-O position, had two metabolites were detected in the purfusate, whereas for epimedin A, epimedin B and epimedin C, which are triglycosides with only one glucose group at 7-O position, one metabolite was formed. From the retention time of these metabolites, it can be deduced that these metabolites are not phase II metabolites, because phase II metabolites would be expected to elute earlier than the intact drug due to its polarity. Apart from baohuoside I, all of the other four prenylated flavonoids have a glucose group at 7-O position, and might be hydrolyzed as secondary glycosides in the intestine, confirming the earlier observation that only the monoglucoside bond is sensitive to the rat intestinal glycosidase [16].

For baohuoside I, in spite of the presence of a monorhamnose bond at the 3-O position, no metabolites were detected, suggesting that the monorhamnose bond could not be hydrolyzed by enzymes in the intestine. Baohuoside I could be absorbed by enterocytes directly, and the amounts absorbed in duodenum and jejunum were much higher (p < 0.05) than in ileum and colon. The permeability of baohuoside I was 2.324 ± 0.410, 2.398 ± 0.143, 1.529 ± 0.339 and 1.045 ± 0.146 in the duodenum, jejunum,ileum and colon, respectively (see Table 1).

**Figure 3:**
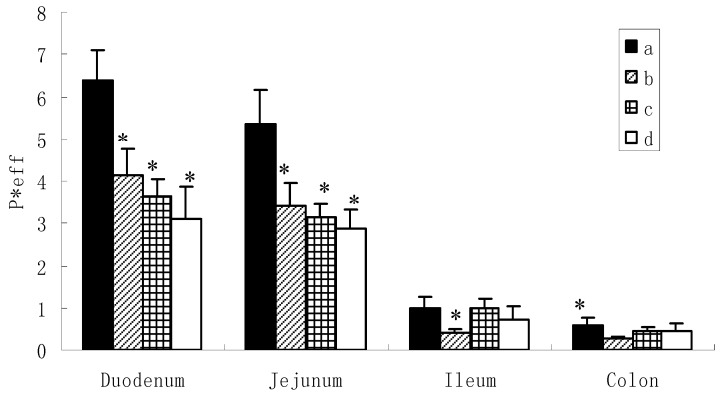
Comparison of permeability of icariin, epimedine A, epimedine B, epimedine C in different intestinal segements. a: icariin, b: epimedin A, c: epimedin B, d: epimedin C. Data are expressed as mean ± SD (n = 4). The statistically significant difference is shown by the asterisk symbol, icariin *vs.* epimedin A, epimedin B, epimedin C, * P < 0.05.

However, the situation was different for the diglycoside and triglycosides (Figure 3). Based on the data obtained (6.315 ± 0.804, 5.227 ± 0.735 for icariin, 4.120 ± 0.629, 3.397 ± 0.567 for epimedin A, 3.645 ± 0.422, 3.135 ± 0.310 for epimedin B and 3.114 ± 0.772, 2.882 ± 0.435 for epimedin C), it appeared that icariin, epimedin A, epimedin B and epimedin C exhibited high permeabilities in the duodenum and jejunum. However, these were not the true permeabilities which could represent the absorbed amounts, because we found large amounts of metabolites coming out at the same time. As aforementioned, these metabolites were unlikely to be phase II metabolites and might be the hydrolysates of the intestinal enzyme. Here, the permeability just represented the disappearance of the amounts of the corresponding compound in intestine, because our previous study has showed that the absorptive permeabilities of these four flavoniods were very low in the intestine [10]. Thus, this disappearance in extracellular concentration provides evidence for extracellular hydrolysis rather than transport across the intestinal mucosa.

It can be observed from Figure 3 that hydrolysis of the flavonoids occurred more rapidly in the duodenum and jejunum than the ileum and colon. This indicated that the intestinal enzyme may distribute more in the upper small intestine, which was consistent with the previous report [17]. Among the four flavonoids, the diglycoside hydrolyzed more readily as can be observed from the higher rate of hydrolysis of icariin (P < 0.05) than epimedin A, epimedin B, and epimedin C in duodenum and jejunum. For the triglycosides, no significant differences were observed among the rate of hydrolysis of epimedin A, epimedin B, and epimedin C in duodenum and jejunum.

For some flavone glucosides, hydrolysis of glucosides to release the aglycones by intestinal glycosidase is a critical first step in their disposition because it serves as the initiator of all subsequent disposition processes. Our data suggest that the intestinal hydrolysis of glycosides is rapid when compared with intestinal transit times of 2 to 4 h. If this hydrolysis in humans occurs as fast as we had observed in the rats, the role played by intestinal microflora in hydrolyzing glycosides could diminish significantly. These results tend to suggest that intestinal glycosidases are brush-border enzymes and LPH is probably the enzyme responsible since it is the only brush-border glycosidase described to date [18], LPH is expressed by humans and rats, with two active site, both identified on the same protein, capable of hydrolysing flavonoid glycosides [19,20]. In the present study, we used gluconolactone, one of the LPH enzyme inhibitor [17], to see if LPH was involved in the hydrolysis of the flavonoids. If LPH plays an important role in the absoption of icariin, epimedin A, epimedin B, and epimedin C, then low activity of LPH would result in a reduced level of metabolites and thereafter a reduced level of them diffusing across the brush-border.

**Figure 4:**
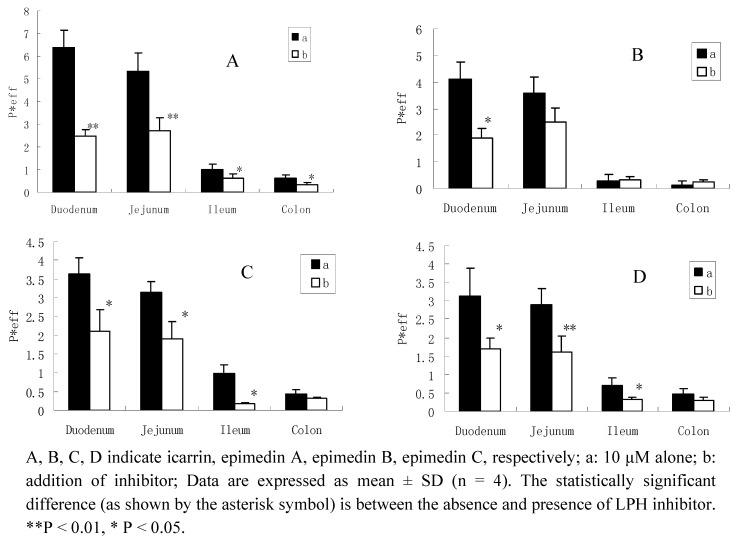
Permeability of 10 μM flavonoids absence or presence of LPH enzyme inhibitor.

When adding gluconolactone, the permeabilities of these four flavonoids decreased in different intestinal segments, especially significantly inhibited in duodenum and jejunum. For icariin, the hydrolysis inhibition rate in the four intestinal segments were 62%(duodenum), 50% (jejunum), 40% (ileum) and 46% (colon), respectively; and also with an inhibition rate of epimedin A, 55%, 26%, 21%,14%; epimedin B, 42%, 40%, 74%, 22%; and epimedin C, 42%, 40%, 52%, 35%, respectively (Figure 4). This evidence confirmed the hypothesis that LPH might be involved in the hydrolysis of the diglycoside and triglycosides.

**Figure 5:**
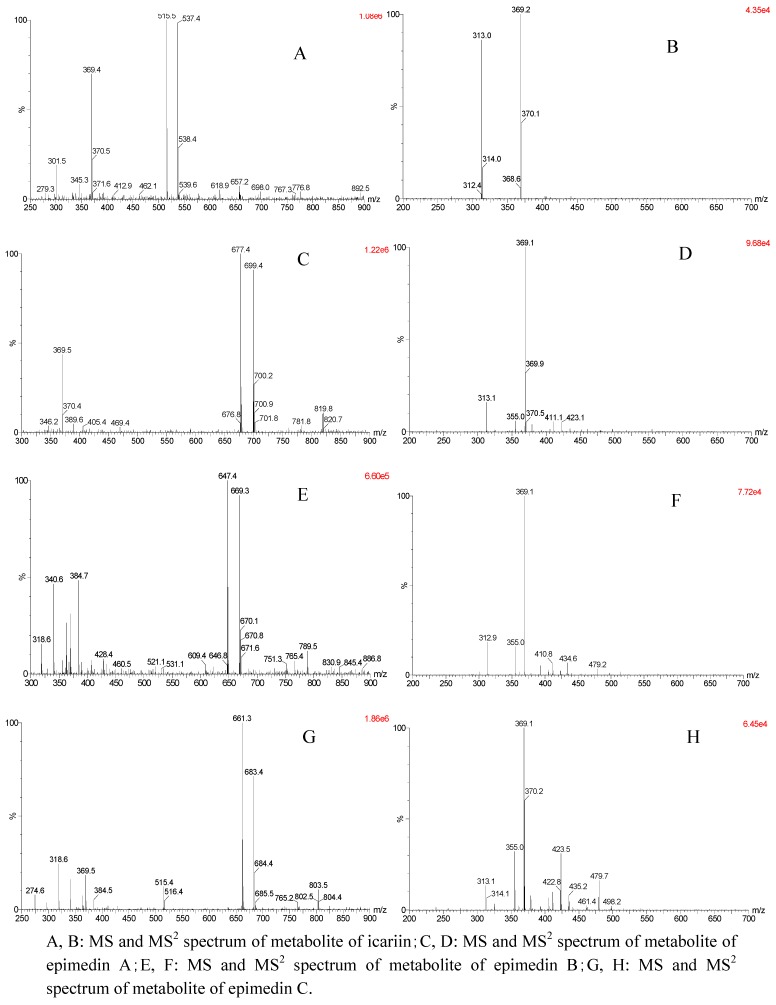
MS^n^ spectra corresponding to the metabolite peaks of prenylflavonoids.

We also used the LC/MS/MS to identify the metabolites of the flavonoids (Figure 5). Characteristic MS/MS product ions were obtained and by measuring mass/charge ratios, it was possible to propose structures for these product ions. In the MS^2^ spectrum of icariin perfusate, the molecule ion [M+H] ^+^ at *m*/*z* 515.5 was a protonated molecular ion and [M+Na] ^+^ ion was at *m*/*z* 537.4. The ion at *m*/*z* 369.4(in the MS^2^ spectrum, it was 369.2) was an aglycon ion cleaved rhamnosyl. The ion at *m*/*z* 313.1[MH^+^-146-56+H] was consistent with the previous report [21], which deduced that one of the metabolites of icariin should be baohuoside I.

Mass data of the epimedin A rat perfusate showed that *m*/*z* 677.4 and 699.4 should be the protonated molecular ion [M+H] ^+^ and [M+Na] ^+^ ion of metabolite of epimedin A, *m*/*z* 369.5 (daughter scan 369.1) should be aglycon, *m*/*z* 423.1 (441.0-18) and *m*/*z* 313.1[(M+H)^+^-162-146-56] was the same as the literature [4,21], which suggests that the metabolite of epimedin A was sagittatoside A. Mass data of the epimedin B rat perfusate showed that *m*/*z* 647.4 [M+H] ^+^and 669.3 [M+Na] ^+^ should be the protonated molecular ion [M+H]^+^ and [M+Na]^+^ ion of metabolite of epimedin B, while *m*/*z* 369.1 should be aglycon, which was the same as the literature [4,21], so it can be deduced that the metabolite of epimedin B was sagittatoside B. Mass data of the epimedin C rat perfusate showed that *m*/*z* 661.3 [M+H] ^+^and 683.4 [M+Na] ^+^should be the protonated molecular ion [M+H] ^+^and [M+Na] ^+^ ion of metabolite of epimedin C, *m*/*z* 369.5 should be aglycon; *m*/*z* 515.4 (M+H^+^-146) and *m*/*z* 313.1[MH^+^-146-146-56] were consistent with the previous report [4,21], which suggested that the metabolite of epimedin C might be 2″-*O*-rhamnosylicariside II.

Based on our analysis and literature data, the absorption pathways of Epimedium flavonoids in the rat intestine initially involved deglycosylation of flavonoid glycosides, yielding the major hydrolytic metabolites. Metabolites were formed by the loss of the saccharide group at C7 via intestinal enzyme. Thus, the metabolite of icariin, epimedin A, epimedin B, and epimedin C were baohuoside I, sagittatoside A, sagittatoside B, and 2″-*O*-rhamnosylicariside II, respectively. These results were consistent with those obtained by Caisheng Wu and co-workers [22], who found that the major metabolite in plasma and bile was baohuoside I, which was the final metabolite of the main flavonoids of *Epimedium koreanum* Nakai, while, in urine and feces, the major metabolites was sagittatoside A (the metabolite of epimedin A) and sagittatoside B (the metabolite of epimedin B). However, most of the previous studies consider that the deglycosylation of flavonoids may be caused by intestinal flora and hepatic biotransformation enzymes [18], but our study demonstrated that intestinal enzyme plays an important role in the absoption of the prenylated flavonoids present in Yinyanghuo, and the 7-O-β glucose was readily hydrolyzed. These evidences confirm that deglycosylation is a prerequisite for absorption of prenylated flavonoids through the small intestine and thereafter secondary glycoside can be transported through highly lipophilic epithelial membrane.

## 3. Experimental

### 3.1. Chemicals

Icariin (purity > 98%) was purchased from National Institute for the Control of Pharmaceutical and Biological Products (China). Epimedin A, epimedin B, epimedin C, and baohuoside I (all > 98% purity) were provided by the Laboratory of Pharmaceutical Preparation (Jiangsu Provincial Academy of Chinese Medicine, China). Gluconolactone (purity > 98%) and Hanks’ balanced salt solution (HBSS; powder form) were purchased from Sigma-Aldrich (St. Louis, MO, USA). Yinyanghuo was supplied from a drug store in Nanjing (China) and was identified to be the correct species (*Epimedium koreamum* Nakai) by Professor Dekang Wu, a pharmacognosy researcher at the Nanjing University of Chinese Medicine (Nanjing, China). All other materials (typically analytical grade or better) were used as received.

### 3.2. Animals

Male Sprague-Dawley rats weighing between 250 and 300 g were obtained from the SLEK Lab Animal Center of Shanghai (Shanghai, China). The rats were fed with water and a standard diet. The rats were fasted overnight before the day of the experiment. No flavonoids were found in pH 7.4 HBSS buffer after perfused through a segment of jejunum, indicating minimal presence of dietary flavonoids in the intestine.

### 3.3. Animal surgery

The surgical procedures were approved by the Animal Ethics Committee of Jiangsu Provincial Academy of Chinese Medicine. Anesthesia was induced by an i.m. injection of urethane (0.5 g/mL). After the rat was anesthetized, it was put over a heating blanket and under a heating lamp to keep its normal body temperature. We then cannulated four segments of the intestine, each with two cannulae. After each cannula was inserted, it was secured with a sterilized black suture before the next cannula was inserted and secured. First, the duodenum was located as the intestinal segment immediately adjacent to the stomach, and two cannulae at 10 cm apart were inserted into two ends of the duodenum and secured with suture. Next, the jejunum was located below the duodenum, and the first cannula was inserted at 4 cm below the duodenal outlet cannula, whereas the second cannula was inserted at 10 cm below the first jejunal cannula. Then, the terminal ileum was located by identifying the ileocecal junction of the rat intestine. The outlet cannula was inserted into the ileum at 2 cm above the junction, and the inlet cannula was inserted 10 cm above the outlet cannula. Lastly, the colon inlet cannula was inserted into the colon at 2 cm below the junction, and the outlet cannula was inserted through the anus. After cannulation, the small intestinal segments were placed carefully into the abdominal cavity, avoiding crimping or kinking of the segments. The incision was then covered by a normal saline-wetted paper towel. A piece of plastic wrap was put on the towel to keep the intestinal segments moist. Additionally, caution was also exercised to keep the inlet and outlet cannulate at the same height to avoid gravitational flow. To keep the temperature of the perfusate constant, the inlet cannulate was kept warm by a 37°C circulating water bath [23].

### 3.4. Transport and metabolism experiments in perfused rat intestinal model

This is a single-pass perfusion method. Four segments of the intestine (duodenum, upper jejunum, terminal ileum, and colon) were perfused simultaneously with a perfusate containing the compound of interest using an infusion pump (Harvard Apparatus, Cambridge, MA, USA) at a flow rate of 0.2 mL/min. After a 30-min washout period, which is usually sufficient to achieve the steady-state absorption, four samples were collected from the outlet cannulae every 30 min afterward. After perfusion, the length of the intestine was measured as described previously [23,24] The outlet concentrations of the test compounds in the luminal perfusate were determined by HPLC or LC/MS/MS.

### 3.5. Enzymatic hydrolysis

Gluconolactone was used as an inhibitor of LPH. 1.78 g gluconolactone was added to the 250 mL of 10 μM icarrin, epimedin A, epimedin B, epimedin C HBSS solution and mixed, respectively. The total gluconolactone concentration was 40μM. After perfusion, perfusates were collected and determined by HPLC.

### 3.6. HPLC analysis of perfusion samples

The conditions for the HPLC analysis of flavonoids in the perfusion samples were as follows: system, Agilent 1100 HPLC with photodiode array detector; column, Zorbax SB-C18, 5 μm, 4.6 × 250 mm (Agilent, Palo Alto, CA, USA); mobile phase A, acetonitrile; mobile phase B, water; gradient, 0~11 min:29% A, 13 min:70% A, 19 min:80% A, 22 min:100% A; flow rate, 1 mL/min; wavelength 268 nm; injection volume, 10 μL. The HPLC chromatograms of perfusate samples are shown in Figure 2. In general, these methods were selective and reproducible with day to day variability of less than 3%. The accuracy and precision were greater than 98%. The tested linear response ranges for all flavonoids were 0.3125 to 40 μM, respectively.

### 3.7. LC/MS/MS identification of metabolites

Different perfusion samples were analyzed by LC/MS/MS using a Waters 2695-Micromass Quattro-micro^TM^ (Waters, Waltham, MA, USA) system equipped with an electrospray ionization source. The samples were separated on a Zorbax SB-C18 column (250 mm × 4.6 mm, 5 μm, Agilent). The mobile phase consisted of acetonitrile (A) and water containing 0.1% acetic acid (B). A gradient program was used as follows: 0~5 min, 29%B, 5~10 min, 29%~90% B, and the flow-rate was 1.0 mL/min. For MS analysis, the electro spray ionization (ESI) source was operated in the positive ion mode. The MS operating parameters were as follows: multireaction monitoring mode (MRM), capillary voltage of 3 Kv, sample cone of 20 V, tube lens offset voltage of 0.1 V, source and desolvation gas temperatures of 120 and 400, cone gas flow rate of 50 L·h^−1^, desolvation gas flow rate of 500 L·h^−1^, collision energy of 20 V, and argon collision gas pressure of 3.85e^−3^.

### 3.8. Data analysis

In the perfused rat intestinal model, *P*_eff_^*^ (effective permeability) is a representation of the intestinal membrane permeability. *P*_eff_^*^ of the compounds are calculated using the following equations:

$${P_{\mathrm{eff}}}^{*}=\frac{1-Cm/Co}{4Gz}$$

where *Co* and *Cm* are inlet and outlet concentrations, respectively; *Gz*, or Graetz number ($Gz=\frac{\pi DL}{2Q}$), is a scaling factor that incorporates flow rate (Q), intestinal length (L), and diffusion coefficients (D) to make the permeability dimensionless. *Cm* was adjusted for water flux, data points were discarded if the water flux exceeded 0.5%/cm of intestine.

### 3.9. Statistical analysis

One-way ANOVA (SPSS 16.0 software) with Tamhane’s post hoc analysis was used to analyze data for multiple comparisons when the variance was shown to be unequal. Unpaired student’s t test (Microsoft Excel) was used to analyze the data when there were only two groups in the experiments. The prior level of significance was set at p < 0.05.

## 4. Conclusions

Prenylated flavonoids present in Yinyanghuo, such as diglycoside (icarrin) and triglycosides (epimedin A, epimedin B, epimedin C) should be hydrolyzed to secondary glycoside prior to absorption in small intestine. Lactase phlorizin hydrolase plays an important role in the hydrolysis of prenylated flavonoids before absorption, and the glucose group at 7-O position was readily to hydrolyze, which would influence the bioavailabilities of these flavonoids later.

## Figures and Tables

**Table 1:** Permeability of baohuoside I in different intestinal segements(mean ± SD, n = 4).

P^*^_eff_	Duodenum	Jejunum	Ileum	Colon
Avg ± sd	2.324 ± 0.410	2.398 ± 0.143	1.529 ± 0.339	1.045 ± 0.146

## References

[1] National Commission of Chinese Pharmacopoeia (2005). Pharmacopoeia of the Peoples Republic of China.

[2] Guo B.L., Xiao P.G. (2003). Comment on main species of Herba Epimedii. Chin. J. Chin. Mater. Med..

[3] Zhang D.W., Cheng Y., Wang N.L., Zhang J.C., Yang M.S., Yao X.S. (2008). Effects of total flavonoids and flavonol glycosides from Epimedium koreanum Nakai on the proliferation and differentiation of primary osteoblasts. Phytomedicine.

[4] Zhao H.Y., Sun J.H., Fan M.X., Fan L., Zhou L., Li Z., Han J., Wang B.R., Guo D.A. (2008). Analysis of phenolic compounds in Epimedium plants using liquid chromatography coupled with electrospray ionization mass spectrometry. J. Chromatogr. A.

[5] Huang X., Zhu D., Lou Y. (2007). A novel anticancer agent, icaritin, induced cell growth inhibition, G1 arrest and mitochondrial transmembrane potential drop in human prostate carcinoma PC-3 cells. Eur. J. Pharmacol..

[6] Nemeth K., Plumb G.W., Berrin J.G., Juge N., Jacob R., Naim H.Y., Williamson G., Swallow D.M., Kroon P.A. (2003). Deglycosylation by small intestinal epithelial cell beta-glucosidases is a critical step in the absorption and metabolism of dietary flavonoid glycosides in humans. Eur. J. Nutr..

[7] Day A.J., Canada F.J., Diaz J.C., Kroon P.A., Mclauchlan R., Faulds C.B., Plumb G.W., Morgan M.R., Williamson G. (2000). Dietary flavonoid and isoflavone glycosides are hydrolyzed by the lactase site of lactase phlorizin hydrolase. FEBS Lett..

[8] Day A.J., Gee J.M., DuPont M.S., Johnson I.T., Williamson G. (2003). Absorption of quercetin-3-glucoside and quercetin-40-glucoside in the rat small intestine: the role of lactase phlorizin hydrolase and the sodium-dependent glucose transporter. Biochem Pharmacol..

[9] Wilkinson A.P., Gee J.M., Dupont M.S., Needs P.W., Mellon F.A., Willlamson G., Johnson I.T. (2003). Hydrolysis by lactase phlorizin hydrolase is the first step in the uptake of daidzein glucosides by rat small intestine *in vitro*. Xenobiotica.

[10] Chen Y., Zhao Y.H., Jia X.B., Hu M. (2008). Intestinal Absorption Mechanisms of Prenylated Flavonoids Present in the Heat-Processed Epimedium koreanum Nakai (Yin Yanghuo). Pharm. Res..

[11] Chantret I., Rodolosse A., Barbat A., Dussaulx E., Brotlaroche E., Zweibaum A., Rousset M. (1994). Differential expression of sucrase-isomaltase in clones isolated from early and late passages of the cell-line Caco-2—evidence for glucose-dependent negative regulation. J. Cell Sci..

[12] Andlauer W., Kolb J., Furst P. (2000). Absorption and metabolism of genistin in the isolated rat small intestine. FEBS Lett..

[13] Andlauer W., Kolb J., Furst P. (2000). Isoflavones from tofu are absorbed and metabolized in the isolated rat small intestine. J. Nutr..

[14] Chen J., Wang S., Jia X., Bajimaya S., Tam V., Hu M. (2005). Disposition of Flavonoids via Recycling: Comparison of Intestinal *versus* Hepatic Disposition. Drug Metab. Dispos..

[15] Ho Y.F., Lai M.Y., Yu H.Y., Huang D.K., Hsueh W.C., Tsai T.H., Lin C.C. (2008). Application of Rat In Situ Single-pass Intestinal Perfusion in the Evaluation of Presystemic Extraction of Indinavir Under Different Perfusion Rates. J. Formos. Med. Assoc..

[16] Liu Y., Dai Y., Xun L., Hu M. (2003). Enteric disposition and recycling of flavonoids and ginkgo flavonoids. J. Altern. Complement. Med..

[17] Liu Z.Q., Jiang Z.H., Liu L., Hu M. (2006). Mechanisms responsible for poor oral bioavailability of paeoniflorin: Role of intestinal disposition and interactionswith sinomenine. Pharm Res..

[18] Day A.J., DuPont M.S., Ridley S., Rhodes M., Rhodes M.J., Morgan M.R., Williamson G. (1998). Deglycosylation of flavonoid and isoflavonoid glycosides by human small intestine and liver beta-glucosidase activity. FEBS Lett..

[19] Lee M.F., Russell R.M., Montgomery R.K., Krasinski S.D. (1997). Total intestinal lactase and sucrase activities are reduced in aged rats. J. Nutr..

[20] Day A.J., Canada F.J., Diaz J.C., Kroon P.A., Mclauchlan R., Faulds C.B., Plumb G.W., Morgan M.R., Williamson G. (2000). Dietary flavonoid and isoflavone glycosides are hydrolyzed by the lactase site of lactase phlorizin hydrolase. FEBS Lett..

[21] Wang Y.Q., Guo Z.M., Jin Y., Zhang X.L., Wang L., Xue X.Y., Liang X.M. (2010). Identification of prenyl flavonoid glycosides and phenolic acids in Epimedium koreanum Nakai by Q-TOF-MS combined with selective enrichment on “click oligo (ethylene glycol)” column. J. Pharm. Biomed. Anal..

[22] Wu C.S., Sheng Y.X., Zhang Y.H., Zhang J.L., Guo B.L. (2008). Identification and characterization of active compounds and their metabolites by high-performance liquid chromatography/Fourier transform ion cyclotron resonance mass spectrometry after oral administration of a herbal extract of Epimedium koreanum Nakai to rats. Rapid Commun. Mass Spectrom..

[23] Hu M., Roland K., Ge L., Chen J., Tyle P., Roy S.J. (1998). Determination of Absorption Characteristics of AG337, A Novel Thymidylate Synthase Inhibitor, Using a Perfused Rat Intestinal Model. Pharm. Sci..

[24] Hu M., Amidon G.L. (1988). Passive and Carrier-Mediated Intestinal Absorption Components of Captopril. J. Pharm. Sci..

